# Targeting the CCL28-STAT3-PLAC8 axis to suppress metastasis and remodel tumor microenvironment in colorectal cancer

**DOI:** 10.3389/fimmu.2025.1610540

**Published:** 2025-10-01

**Authors:** Yao Yang, Qixin Jiang, Zhe Zhu, Shun Zhang, Tao Du, Shuzheng Song, Xiaohua Jiang

**Affiliations:** ^1^ Department of Colorectal Surgery, Department of General Surgery, Shanghai East Hospital, School of Medicine, Tongji University, Shanghai, China; ^2^ Department of Gastrointestinal Surgery, Shanghai East Hospital, School of Medicine, Tongji University, Shanghai, China

**Keywords:** PLAC8, colorectal cancer, tumor microenvironment, EMT, CCL28

## Abstract

**Background:**

Chronic inflammation plays a critical role in the initiation and progression of colorectal cancer (CRC), establishing a close link between the inflammatory microenvironment with tumor invasion and metastasis. However, the regulatory mechanisms by which inflammation-related factors promote CRC progression remain largely unclear.

**Methods:**

The biological significance of PLAC8 in colorectal cancer was investigated through clinical data analysis, mouse models of colitis-associated colorectal cancer, gene knockdown and overexpression, as well as cell migration and invasion assays. Additionally, bioinformatics analysis, activation and inhibition of PI3K/Akt and JAK/STAT3 signaling pathways, along with techniques including CUT&Tag, Western blotting, and qPCR, were employed to comprehensively analyze the detailed molecular mechanisms of PLAC8.

**Results:**

Analysis of PLAC8 expression in 78 paired clinical samples revealed significantly elevated PLAC8 expression in CRC and was identified as an independent prognostic factor. Increased expression of PLAC8 was further validated in the mouse inflammation-cancer transition model. Genetic manipulation of PLAC8 through overexpression and knockdown unequivocally established its prometastatic function in CRC, with no significant effects on proliferation, oxaliplatin resistance, or colony formation. Pharmacological modulation of AKT signaling using specific activators (SC79) and inhibitors (Capivasertib) confirmed that PLAC8 drives EMT through AKT pathway activation, resulting in increased expression of EMT-related proteins, such as N-cadherin and Snail, thereby enhancing cell migration and invasion. Further correlation analysis, CUT&Tag, and STAT3 inhibition studies revealed that CCL28 activated the STAT3 signaling pathway, promoting PLAC8 expression, and ultimately enhancing CRC invasion and metastasis.

**Conclusion:**

CCL28-mediated promotion of PLAC8 via the JAK/STAT3 signaling pathway, led to EMT in colorectal cancer cells, which played a key role in the transition from inflammation to cancer. PLAC8 served as an independent risk factor for colorectal cancer prognosis.

## 1 Introduction

In recent years, the incidence of colorectal cancer (CRC) has increased dramatically, largely attributable to changes in dietary habits and lifestyle. According to the most recent global epidemiological data, a total of 1,926,118 new CRC cases were diagnosed worldwide in 2022, positioning it as the third most common cancer. Furthermore, CRC was responsible for 903,859 cancer-related deaths, making it the second leading cause of cancer mortality globally, surpassed only by lung cancer (1). In China, 517,100 new cases of CRC were reported in 2022, ranking second among all cancers, while CRC-related deaths reached 240,000, placing it fourth overall and second among females (2). CRC imposes not only a significant physical and psychological burden on patients and families, but also a considerable economic strain on society.

Inflammation plays a crucial role in the onset and progression of cancer, serving as a significant driver in tumor initiation and advancement (3, 4). The relationship between inflammation and cancer has been a major focus of research. Numerous epidemiological studies have confirmed that inflammatory bowel diseases, such as ulcerative colitis and Crohn’s disease, significantly increase the risk of developing colorectal cancer (5, 6). The highly dynamic and complex inflammatory tumor microenvironment (TME) (7, 8), comprising key inflammatory mediators such as tumor-associated macrophages (TAMs), tumor-associated neutrophils (TANs), dendritic cells (DCs), myeloid-derived suppressor cells (MDSCs), and T lymphocytes (9), plays a pivotal role in tumor progression. Inflammatory cytokines like TNF-α, TGF-β, IFN-γ, IL-1, IL-6, and IL-10 (10), along with their downstream intracellular signaling pathways, including eicosanoid signaling and the Janus kinase (JAK)-signal transducer and activator of transcription (STAT) pathway, are critical to tumorigenesis. Targeting inflammation through modulation of these mediators and signaling pathways holds therapeutic promise by modulating the tumor microenvironment, thereby inhibiting tumor growth and progression (11, 12). However, the molecular mechanisms linking inflammation and colorectal cancer development remain incompletely defined.

Placenta-specific gene 8 (PLAC8), alternatively known as Onzin or C15, is a conserved cysteine-rich protein expressed across eukaryotic species. It is predominantly expressed in various immune cells and tumor cells. Studies have shown that abnormal expression of PLAC8 in monocytes is associated with inflammatory storms in conditions such as sepsis and COVID-19 (13–16). In IBD patients, elevated PLAC8 expression in the gut microbiome correlate with dysbiosis and may serve as a microbial biomarker predicting higher colorectal or gastric cancer risk (17). Furthermore, PLAC8 has been implicated in the initiation and progression of several types of cancer, including breast cancer, liver cancer, and colorectal cancer, where it plays a role in tumor cell growth, invasion, metastasis, and apoptosis, among other processes (18). Some studies have also found that PLAC8 expression is decreased in colorectal cancer tissues, and it acts as a tumor suppressor gene by inhibiting the immune response (19, 20). However, the precise role of PLAC8 in the inflammation-associated carcinogenesis (inflammation-cancer transition) of colorectal cancer, and its underlying molecular mechanisms remain incompletely understood.

Preliminary findings suggest that the PLAC8 gene is involved in the inflammation-cancer transition in colorectal cancer, although its precise mechanism requires further investigation. Therefore, this study aimed to explore the role of PLAC8 in the pathogenesis and progression of colorectal cancer, with a focus on the inflammation-cancer transition. We employed a combination of animal models, bioinformatics approaches, and molecular biology techniques to investigate how PLAC8 is intricately involved in colorectal cancer development and assess its potential as a novel therapeutic target.

## 2 Materials and methods

### 2.1 Colorectal cancer clinical case data collection

Seventy-eight CRC patients with complete follow-up and clinical data treated at our institution were enrolled, and data collected included age, gender, clinicopathological characteristics (TNM stage, differentiation grade), tumor location, and serum tumor markers (CEA, CA19-9, CA125). Five cases without paired normal tissues were excluded from tumor-normal IHC comparisons, and three cases with missing CEA/CA199 values were omitted from regression analyses. Written informed consent forms were obtained from all participants. All procedures were approved by the Institutional Review Board (IRB) and the Medical Ethics Committee of Shanghai East Hospital.

### 2.2 Immunohistochemistry

Immunohistochemical (IHC) staining was performed to assess PLAC8 expression in CRC and adjacent normal tissues. Paraffin-embedded tissue sections (4 μm thick) were deparaffinized in xylene and rehydrated through a graded ethanol series. Antigen retrieval was carried out using citrate buffer (pH 6.0) in a microwave for 10 minutes. Upon cooling to ambient temperature, endogenous peroxidase activity was quenched with 3% hydrogen peroxide for 10 minutes. The sections were then incubated with a primary anti-PLAC8 antibody (Proteintech, 12284-1-AP, 1:100) overnight at 4 °C, followed by a 30-minute incubation at room temperature with a biotinylated secondary antibody. Signals were detected using a DAB substrate kit, and sections were counterstained with hematoxylin. Stained slides were dehydrated, mounted, and examined under a light microscope. PLAC8 expression was evaluated based on staining intensity and the percentage of positive cells. Statistical analysis was conducted to compare PLAC8 expression levels between tumor and adjacent normal tissues. The immunohistochemical (IHC) scoring: Staining intensity was scored as follows: 3 (high), 2 (moderate), 1 (low), and 0 (no). The proportion of positive tumor cells: <10% was scored as 0, 10–25% as 1, 25–50% as 2, 50–75% as 3, and >75% as 4. The final IHC score was calculated by multiplying the intensity score by the proportion score, with the total score used for statistical analysis.

### 2.3 Construction of enteritis and colorectal cancer model

**Figure 1:**
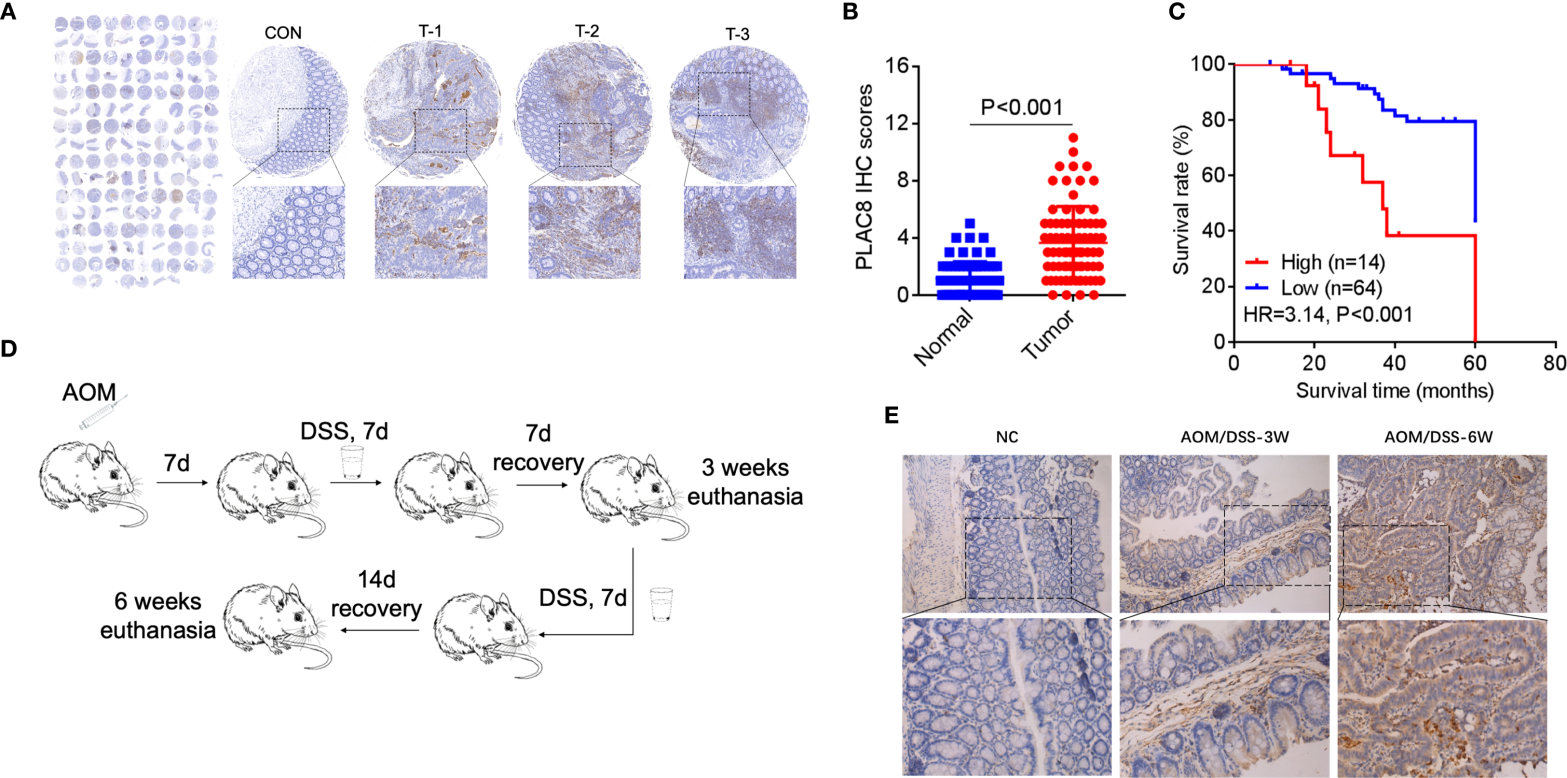
**(A)** PLAC8 IHC in tissue microarray (78 tumor/normal pairs). CON: normal adjacent tissue; T-1/T-2/T-3: representative PLAC8 protein highly expression tumor tissues. **(B)** Comparative IHC scores between tumor and normal tissues. **(C)** Patients were stratified into subgroups based on PLAC8 IHC scoring criteria and survival status, followed by Kaplan-Meier survival curve analysis with log-rank testing. **(D)** The colorectal cancer chemoprevention model was established through sequential administration of azoxymethane (AOM) and dextran sulfate sodium (DSS) in C57BL/6 mice. **(E)** PLAC8 protein expression dynamics were analyzed throughout the tumorigenesis process. (Student’s t-test for comparisons).

The azoxymethane (AOM)/dextran sulfate sodium (DSS) model of colitis-associated colorectal cancer was utilized into the experiment. 8-week-old female C57BL/6 mice (Beijing Vital River) were used. All experimental groups were balanced for age and sex. Animal grouping: control group (CON), experimental control group (DSS), experimental group (AOM/DSS, AD). Ten C57BL/6 mice were assigned to each group. The control group received no treatment, while mice in the experimental group were intraperitoneally injected with 12.5 mg/kg of AOM (MilliporeSigma, A5486). Normal drinking water was provided. One week later, the mice were given 2.5% DSS (Selleck, S6929) in drinking water for 7 consecutive days, with the DSS solution replaced every 2 days. Normal drinking water was then restored for 7 days, constituting one 3-week DSS cycle. Subsequently, mice received 2.5% DSS for 7 consecutive days, followed by a 14-day drug withdrawal period, which constituted the second DSS cycle. CON, DSS and AD groups (n=5/timepoint) underwent synchronized euthanasia at matched timepoints (3 and 6 weeks) (**Figure 1C**). The animal experiments were reviewed and approved by the Institutional Animal Care and Use Committee (IACUC) and Institutional Ethics Committee at Shanghai East hospital.

### 2.4 Cell lines and reagents

Cell lines were obtained from the American Type Culture Collection (ATCC). Cancer cell lines were cultured in RPMI-1640 supplemented with 10% FBS and 1% penicillin–streptomycin, and maintained at 37 °C in a humidified incubator at 5% CO2. SC79, Capivasertib, Ruxolitinib, recombinant CXCL1 and recombinant CCL28 were purchased from Selleck Chemicals and MedChemExpress (S7863, S8019, S1378, HY-P70508, HY-P7250). Ruxolitinib: 2 μM, 24h; SC79: 10 μg/mL, 24h; Capivasertib: 1 μM, 24h. Doses were based on prior literature. Antibodies against p-AKT, p- NF-κB, p-STAT3, STAT3, AKT, E-cadherin, N-cadherin, Snail and GAPDH were purchased from Cell Signaling Technology (CST) (Cat.4060, 3033, 9145, 9135, 9272, 3195, 4061, 3879, 2118). The eukaryotic expression plasmid pcDNA3.1-pGFP-PLAC8 and siRNAs were purchased from GeneChem (Shanghai). Plasmid and siRNAs transfection was performed using Lipofectamine 3000 (Invitrogen, USA). Plasmid and siRNAs were transiently transfected into cells, and subsequent experiments were performed 48 hours post-transfection following validation at both RNA and protein levels. The sequences of primers are listed in **Supplementary Table S1**.

### 2.5 CUT & Tag assay

Targeted CUT&Tag-qPCR was performed to validate STAT3 binding to specific sites within the PLAC8 promoter. Genome-wide sequencing was not conducted due to the focused hypothesis-driven scope of this study. HCT116 cells were permeabilized and incubated with an anti-STAT3 primary antibody, followed by a secondary antibody conjugated to Tn5 transposase. Tagmentation buffer was added to cleave and tag DNA regions bound by STAT3. DNA was extracted and amplified using primers specific for the PLAC8 promoter region (Primer_1 and Primer_2). PCR products were analyzed via agarose gel electrophoresis. Enrichment in the PLAC8 promoter region was quantified by qPCR relative to an IgG control. Primers flanking STAT3-binding motifs at chr4:83115654-83115749 (Primer_1) and chr4:83115648-83115729 (Primer_2) were in the promoter region of PLAC8. The sequences of primers are listed in **Supplementary Table S1**.

### 2.6 Western blot and qPCR

Cellular proteins were extracted in RIPA buffer followed by protein concentration quantification via BCA assay. Equal amounts of protein (20-40 µg) were separated by SDS-PAGE on a 10-15% gel and transferred to a PVDF membrane. The membrane was blocked with 5% non-fat milk in TBST for 1 hour and incubated with primary antibodies overnight at 4 °C, followed by washing with TBST. After incubation with HRP-conjugated secondary antibody, proteins were detected using ECL substrate. For qPCR, total RNA was extracted and cDNA was synthesized from 1 µg of RNA. qPCR was performed using SYBR Green, cDNA, and gene-specific primers, with amplification conditions of 95 °C for 3 minutes, followed by 40 cycles of 95 °C for 10 seconds and 60 °C for 30 seconds. Gene expression was analyzed using the ΔΔCt method and normalized to housekeeping genes. The sequences of primers are listed in **Supplementary Table S1**.

### 2.7 Cell proliferation, clone formation and oxaliplatin sensitivity assay

Following transfection with siRNA or overexpression plasmids, cells were incubated for 48 hours before proceeding to subsequent cell proliferation and colony formation assays. Cell proliferation was assessed using the CCK-8 assay. Cells were seeded into 96-well plates at a density of 2 × 10^3 cells per well and cultured overnight to facilitate attachment. After treatment, 10 µL of CCK-8 solution (Dojindo, CK04) was added to each well, and cells were incubated for 1–4 hours at 37 °C. The absorbance at 450 nm was measured using a microplate reader to assess cell viability (BioTek Synergy H1). The relative cell proliferation rate was calculated by comparing the absorbance of treated samples to that of the control group. For the colony formation assay, cells were seeded into six-well plates at a density of 1,000 cells per well and cultured for 14 days to form colonies. The medium was replaced every 3–4 days. After incubation, cells were fixed with 4% paraformaldehyde for 15 minutes and stained with 0.1% crystal violet for 30 minutes at room temperature. Colonies containing more than 50 cells were counted under a microscope. The colony formation efficiency was calculated by dividing the number of colonies by the number of seeded cells, and the results were compared between different treatment groups. For the oxaliplatin sensitivity assay, cells were seeded in 96-well plates at a density of 3×10³ cells/well and cultured for 24 hours. Oxaliplatin (Sigma-Aldrich, O9512) was serially diluted to create concentration gradients (0, 1, 2, 4, 8, 16, 32 μM) in complete medium. After 48-hour drug exposure, 10 μL CCK-8 reagent was added per well followed by 2-hour incubation at 37 °C. Absorbance at 450 nm was measured using a microplate reader. Cell viability was calculated as: (OD_treatment - OD_blank)/(OD_control - OD_blank) ×100%. Dose-response curves were generated through nonlinear regression analysis (four-parameter logistic model) in GraphPad Prism 9.0 to determine IC50 values. Three independent biological replicates, each with six technical replicates, were performed.

### 2.8 Immigration and invasion assay

The Transwell assay was used to assess cell migration and invasion. For migration, 5 × 10^4 cells were suspended in serum-free medium and seeded into the upper chamber of Transwell insert an 8-µm pore size Transwell insert, while the lower chamber was filled with complete medium containing 10% FBS as a chemoattractant. For invasion, the upper chamber was pre-coated with Matrigel to mimic the extracellular matrix. After incubation for 48h at 37 °C, non-migrated or non-invaded cells on the upper surface of the membrane were removed using a cotton swab. Cells that had migrated or invaded to the lower surface were fixed with 4% paraformaldehyde and stained with 0.1% crystal violet. The number of migrated or invaded cells was counted under a microscope in at least five randomly selected fields. Results are presented as the mean number of migrated/invaded cells in the experimental group relative to the control group indicating the effects of different treatments on cell migration and invasion ability.

### 2.9 Enrichment and correlation analysis based on the TCGA database

Enrichment and correlation analyses were performed using transcriptomic data from the TCGA colorectal cancer dataset. RNA-seq data and corresponding clinical information were obtained from the Genomic Data Commons (GDC) portal. Data preprocessing was performed to filter low-expressed genes and normalize expression values (TPM or FPKM) using the R package TCGAbiolinks. Gene Set Variation Analysis (GSVA) was then applied to evaluate the enrichment of predefined gene sets across samples. For correlation analysis, Pearson correlation coefficients were calculated to assess the association between PLAC8 expression and inflammatory pathway-related gene expression.

### 2.10 Statistics

All experiments included ≥3 biological replicates with ≥2 technical replicates each. Data are presented as mean ± 95% confidence interval (CI) from three independent experiments. Pearson correlation analysis of PLAC8 and inflammatory pathway-related genes was performed. The student’s t-test was performed in GraphPad Prism 6.0 (GraphPad Prism) unless otherwise specified. Variance similarity was assumed between compared groups. Correlation matrices were visualized using hierarchical clustering implemented in the R “corrplot” package. Survival analysis, including Kaplan-Meier curve generation, log-rank tests, and univariate and multivariate Cox regression analyses, was performed using the R “survival” package. P-values < 0.05 were considered statistically significant.

## 3 Results

### 3.1 PLAC8 expression played a crucial role in colorectal cancer and closely associated with survival and prognosis

Seventy-eight CRC patients were enrolled. Clinical data including age, gender, TNM stage, tumor differentiation grade, and serum levels of CEA, CA19-9, and CA125 were collected and analyzed (**Supplementary Table S2**). In three cases, CA19–9 and CEA data were missing. A total of 78 colorectal cancer tissues and 73 paired adjacent non-cancerous tissues underwent immunohistochemical (IHC) analysis to assess PLAC8 protein expression. IHC staining intensity was systematically scored, and comparative analysis between cancerous and adjacent tissues revealed significantly higher PLAC8 expression levels in colorectal cancer tissues compared to adjacent non-cancerous counterparts (P<0.001) (**Figures 1A, B**). ROC curve analysis based on PLAC8 IHC scores and patient survival status determined an optimal cutoff value of 5.5. Patients were stratified into low-expression (<5.5, n=64) and high-expression (≥5.5, n=14) groups. Kaplan-Meier analysis coupled with log-rank testing revealed significantly reduced overall survival in the PLAC8 high-expression group compared to the low-expression group (HR = 3.14, 95% CI 1.72-5.73; P<0.001) (**Figure 1C**).

Analysis of clinicopathological characteristics stratified by PLAC8 expression levels revealed that the high-expression group had a significantly higher proportion of patients with elevated CA19–9 levels compared to the low-expression group (P = 0.008). No significant differences were observed in other demographic, clinical, or histopathological parameters (all P>0.05) (**Supplementary Table S3**). Univariate Cox regression analysis identified significant prognostic associations for T stage (P = 0.001), N stage (P<0.001), M stage (P<0.001), TNM stage (P<0.001), tumor differentiation grade (P = 0.006), CA19–9 levels (P = 0.021), and PLAC8 expression (P = 0.021) (**Supplementary Table S4**). Subsequent multivariate Cox regression analysis identified TNM stage (HR = 3.38, P<0.001), tumor differentiation grade (HR = 2.43, P = 0.01), and PLAC8 expression (HR = 2.87, P = 0.015) as independent risk factors for colorectal cancer prognosis (**Table 1**).

**Table 1:** Cox multivariate analysis of clinicopathological characteristics and PLAC8 expression.

Variables	Cases (n)	HR	*P* value
TNM staging		3.38	<0.001
I	10		
II	17		
III	32		
IV	16		
Differentiation		2.43	0.01
High	1		
Moderate	50		
Low	24		
CA199		1.49	0.29
≥37U/mL	23		
<37U/mL	52		
PLAC8		2.87	0.015
High	13		
Low	62		

### 3.2 Establishment of AOM/DSS animal model and cell models in colorectal cancer

The colitis-associated colorectal cancer mouse model was successfully established using azoxymethane (AOM) combined with dextran sulfate sodium (DSS) (**Supplementary Figure S1**). Immunohistochemical staining revealed that, at 3 weeks (first cycle, n=6) and 6 weeks (second cycle, n=6) confirmed successful model establishment and revealed a significantly higher percentage of PLAC8-positive area from the tumor tissues (relative to the total pathological tissue section area) in comparison to the control group (n=4) and DSS group (n=4) (P<0.05) (**Figures 1D, E**).

**Figure 2:**
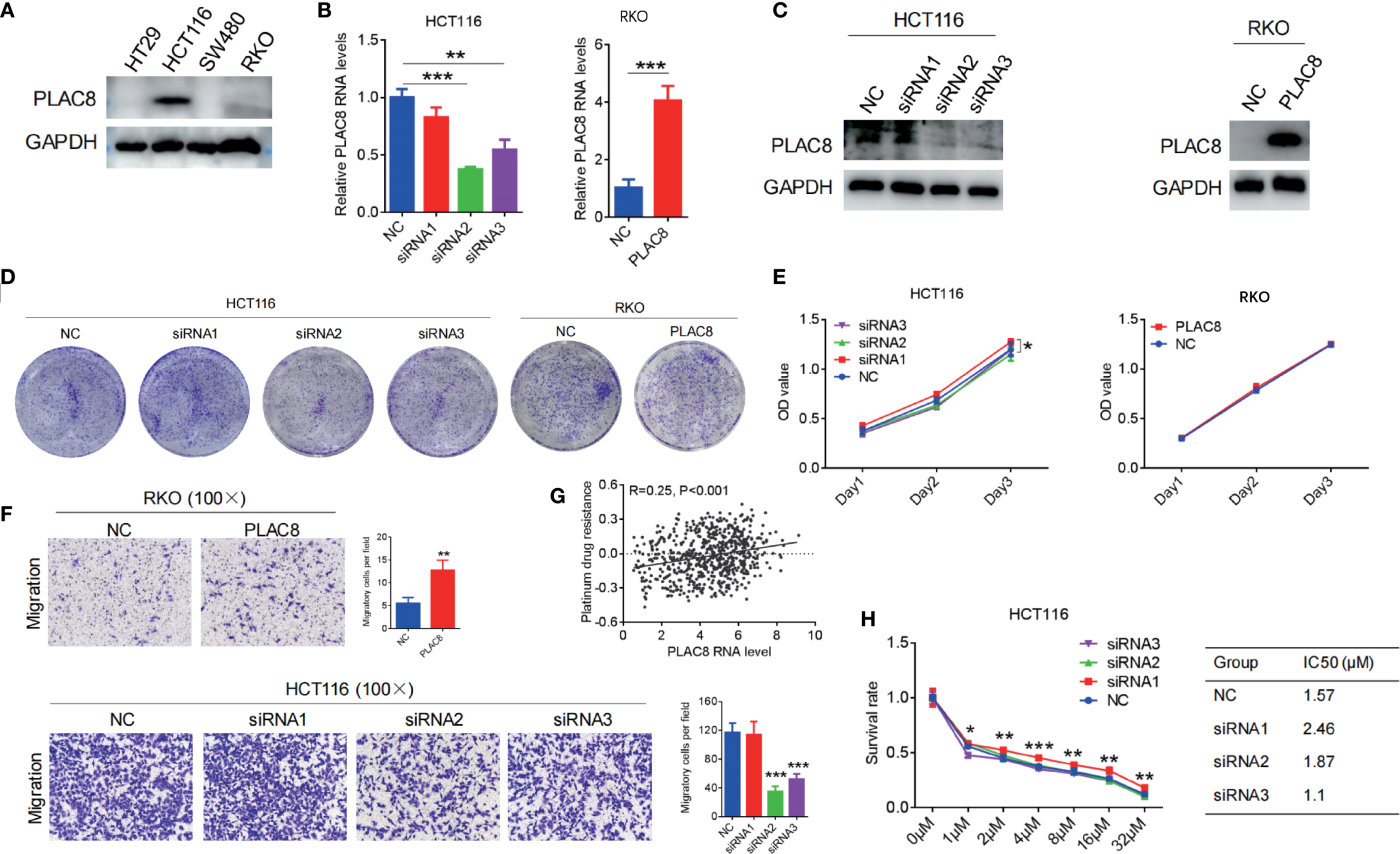
**(A)** PLAC8 expression in four CRC cell lines. **(B, C)** PLAC8 overexpression and knockdown conducted in lower and higher PLAC8 expression CRC cell lines. **(D, E)** Impact of PLAC8 expression changes on cell clonogenicity and proliferation. **(F)** Transwell assay assessing PLAC8 expression on migration of CRC cell lines. **(G)** TCGA-based bioinformatic analysis to explore PLAC8 involvement in platinum resistance. **(H)** Impact of PLAC8 expression changes on oxaliplatin resistance (Student’s t-test for comparisons; *, **, and *** represent P < 0.05, P < 0.01, and P < 0.001, respectively; GSVA for enrichment).

PLAC8 protein expression levels were examined in colorectal cancer cell lines HT29, HCT116, SW480, and RKO. Among CRC cell lines, PLAC8 expression was highest in HCT116, lower in RKO, and undetectable in HT29 and SW480 cells (**Figure 2A**). Therefore, HCT116 cells were used for siRNA knockdown experiments, while RKO cells were selected for PLAC8 overexpression experiments. Western blot and qPCR analysis showed that siRNA1 had a minimal effect on PLAC8 expression, whereas siRNA2 and siRNA3 significantly reduced PLAC8 expression (**Figures 2B, C**). After PLAC8 overexpression, both protein and mRNA levels of PLAC8 were significantly elevated (**Figures 2B, C**).

### 3.3 The impact of PLAC8 gene regulation on the biological functions of colorectal cancer cell lines

Gene knockdown and overexpression of PLAC8 in HCT116 and RKO colorectal cancer cell lines showed that PLAC8 expression did not significantly affect cell proliferation, colony formation (P>0.05) (**Figures 2D, E**). GSVA of TCGA CRC transcriptomes revealed that PLAC8 gene expression was positively correlated with platinum resistance (R = 0.25, P<0.001) (**Figure 2G**). However, after downregulating PLAC8 expression in HCT116, there was no significant difference in the half-maximal lethal dose of oxaliplatin among the groups (P>0.05) (**Figure 2H**). But, siRNA-mediated knockdown of PLAC8 in HCT116 cells significantly reduced cell migration (P<0.001), while PLAC8 overexpression in RKO cells led to a marked increase in cell migration (P<0.01) (**Figure 2F**).

### 3.4 AKT signaling and EMT mediate as critical mediators of PLAC8’s biological functions

**Figure 3:**
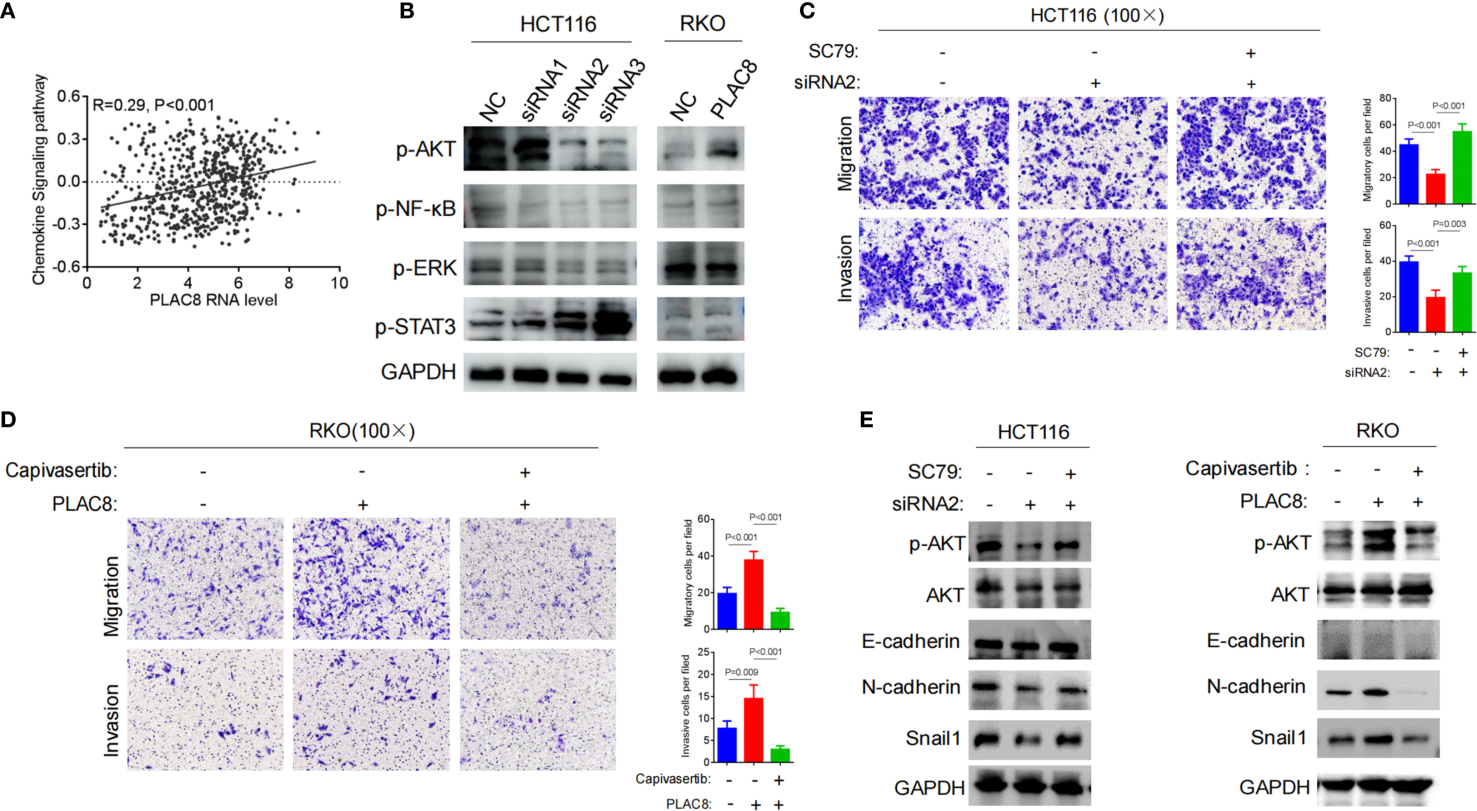
**(A)** TCGA-based bioinformatic analysis to explore PLAC8 involvement in chemokine signaling. **(B)** Effect of PLAC8 expression changes on inflammation and cytokine-related signaling pathways. **(C)** Reversal by AKT pathway activator SC79, the reduction in migration and invasion abilities induced by PLAC8 knockdown. **(D)** Reversal by AKT inhibitor capivasertib, the enhanced migration and invasion abilities of colorectal cancer cells induced by PLAC8 overexpression. **(E)** Effects of PLAC8 modulation and AKT pathway activator and inhibitor on EMT-related protein expression. (GSVA for enrichment).

We also analyzed the potential functional pathways of the PLAC8 gene based on TCGA transcriptomic data, and the results showed a positive correlation with the chemokine signaling pathway (R = 0.29, P<0.001) (**Figure 3A**). Western blot analysis of classical inflammation-related signaling pathways, including AKT, NF-κB, ERK, and STAT3, revealed that siRNA-mediated PLAC8 knockdown significantly reduced p-AKT levels, while p-STAT3 levels were markedly elevated. No significant changes were observed in p-NF-κB and p-ERK levels. In contrast, PLAC8 overexpression led to a significant increase in p-AKT levels, with no notable changes in p-STAT3, p-NF-κB, or p-ERK levels (**Figure 3B**).

After knockdown of PLAC8 expression using siRNA2 in HCT116 cells, migration and invasion were significantly reduced (P<0.001, P<0.001). However, when the AKT signaling pathway activator SC79 was added, cell migration and invasion significantly increased (P<0.001, P = 0.003) (**Figure 3C**). Similarly, PLAC8 overexpression in RKO cells led to increased cell migration and invasion (P<0.001, P = 0.009), while subsequent treatment with the AKT pathway inhibitor capivasertib significantly reduced cell migration and invasion (P<0.001, P<0.001) (**Figure 3D**).

After siRNA2-mediated PLAC8 knockdown in HCT116 cells, protein levels of p-AKT, N-cadherin, and Snail1 were significantly reduced, while the addition of the AKT signaling pathway activator SC79 resulted in increased in these protein levels (P<0.001, P = 0.003). However, E-cadherin levels did not show significant changes (**Figure 3E**). Similarly, after overexpressing PLAC8 in RKO cells, p-AKT, N-cadherin, and Snail1 protein levels were significantly elevated, and following treatment with the AKT pathway inhibitor capivasertib, these levels decreased significantly. E-cadherin levels remained unchanged (**Figure 3E**).

### 3.5 CCL28-activated STAT3 signaling drives PLAC8 transcription to promote colorectal cancer progression

**Figure 4:**
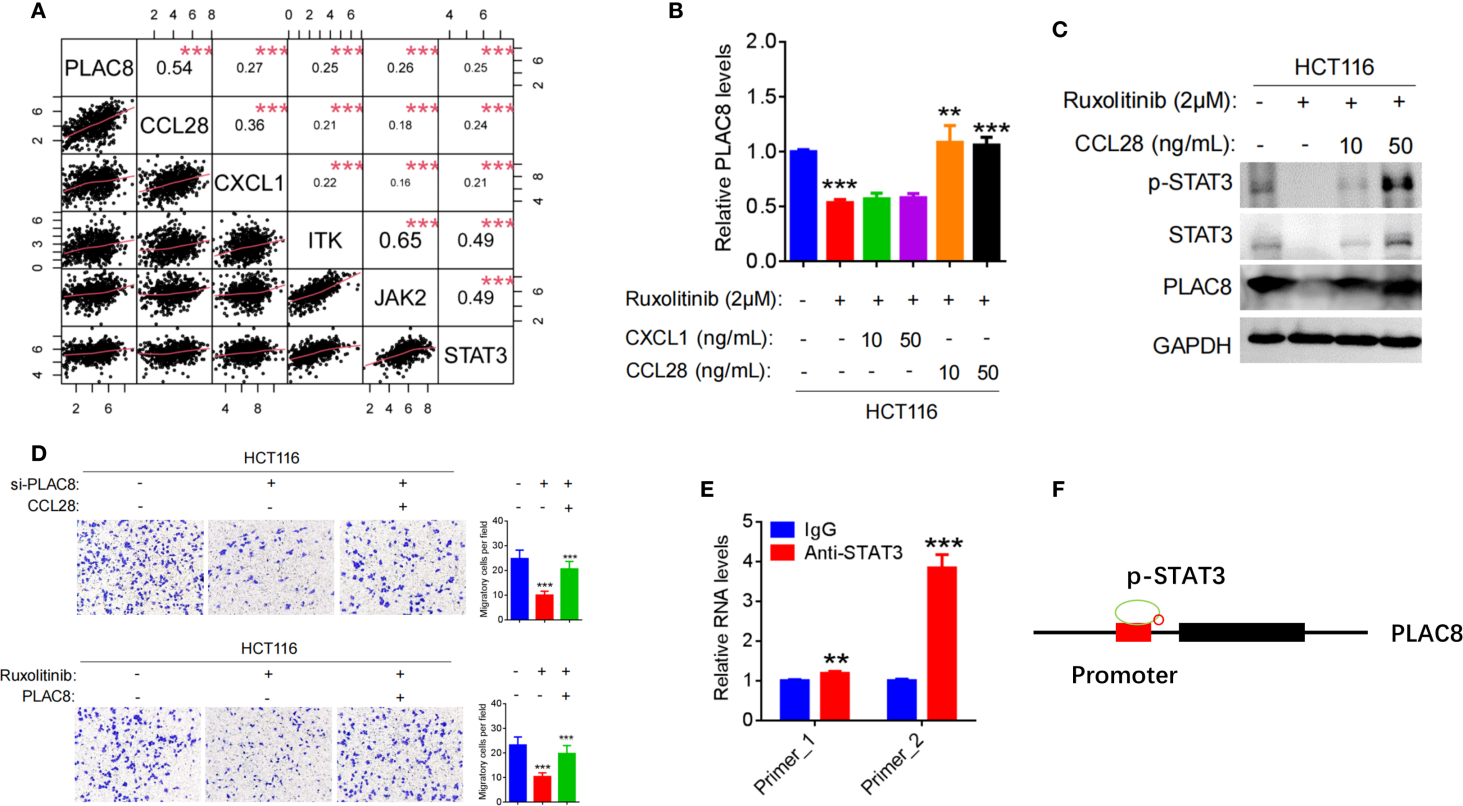
**(A)** Pearson correlation analysis between PLAC8 expression and the top five inflammatory cytokine pathways gene based on the TCGA database. **(B)** Regulation of PLAC8 mRNA expression by Ruxolitinib (STAT3 inhibitor), CXCL1, and CCL28. **(C)** Regulation of STAT3 signaling and PLAC8 protein expression by Ruxolitinib and CCL28. **(D)** Effects of combined PLAC8 knockdown and CCL28 treatment, or STAT3 inhibition and PLAC8 overexpression, on CRC cell migration. **(E, F)** Validation of STAT3 binding to the PLAC8 promoter by CUT&Tag. (**, and *** represent P < 0.01, and P < 0.001, respectively).

Analyses of the TCGA database guided the research direction and hypothesis formulation in this study. Integrative bioinformatics interrogation revealed significant positive correlations between PLAC8 expression and key inflammatory mediators, including CCL28, CXCL1, ITK, JAK2, and STAT3 (correlation coefficients: 0.54, P = 8.25×10^−49^; 0.27, P = 4.10×10^−12^; 0.25, P = 1.02×10^−10^, 0.26, P = 6.05×10^−11^; 0.25, P = 1.68×10^−10^ respectively) (**Figure 4A**).

After treating HCT116 cells with the JAK/STAT3 inhibitor Ruxolitinib, PLAC8 expression was significantly reduced (P<0.001). Upon addition of the recombinant cytokine CCL28, PLAC8 expression was significantly elevated (P<0.01; P<0.001), whereas no significant change in PLAC8 expression was observed upon adding the recombinant chemokine CXCL1 (P>0.05) (**Figure 4B**). p-STAT3 expression exhibited a similar pattern to PLAC8 (**Figure 4C**).

After PLAC8 knockdown with siRNA2 in HCT116 cells, cell invasion was significantly reduced (P<0.001). However, when CCL28 was added, cell invasion significantly increased (P<0.001). Conversely, after PLAC8 overexpression in RKO cells, cell invasion increased (P<0.001), and addition of CCL28 resulted in a marked increase in invasion (P<0.001). After treatment with the JAK/STAT3 inhibitor Ruxolitinib, HCT116 cell invasion decreased significantly (P<0.001). However, PLAC8 overexpression after Ruxolitinib treatment led to a significant increase in cell invasion (P<0.001) (**Figure 4D**).

CUT&Tag experiment was performed using STAT3 antibody was performed to assess STAT3 binding enrichment in the PLAC8 promoter region. Two pairs of primers (Primer_1 and Primer_2) were used to detect DNA enrichment. Primers flanking STAT3-binding motifs at chr4:83115654-83115749 (Primer_1) and chr4:83115648-83115729 (Primer_2) showed 1.19-fold (P = 0.0037) and 3.84-fold (P < 0.001) enrichment vs. IgG. (**Figure 4E**).

## 4 Discussion

In recent years, the role of inflammation in CRC has received increasing attention. Chronic inflammation is recognized not only as a driving factor in the initiation and progression of CRC, but also plays a key role in tumor invasion, metastasis, and resistance to treatment (11).The inflammatory tumor microenvironment in CRC is highly complex, involving various immune cells, inflammatory mediators, and cytokines. Tumor-associated macrophages (TAMs), tumor-associated neutrophils (TANs), and other immune cells secrete pro-inflammatory cytokines (such as TNF-α, IL-6, and IL-1β) that activate signaling pathways like NF-κB and JAK/STAT3, promoting tumor cell proliferation, migration, metastasis, and epithelial-mesenchymal transition (EMT) (6). These immune populations not only fuel tumor progression but also orchestrate immune evasion. To investigate the mechanistic role of inflammation in CRC, various animal models have been developed to simulate this process. The AOM/DSS model is among the most widely used models in inflammation-cancer transition research. AOM is a known colorectal carcinogen, and DSS is a chemical agent that induces colitis. The combination of AOM and DSS is a commonly used model for studying inflammation-induced carcinogenesis in CRC. AOM induces DNA damage and gene mutations in colonic epithelial cells, while DSS induces chronic inflammation in the gut. DSS disrupts the intestinal barrier, promotes intestinal inflammation, and exacerbates the carcinogenic process. In this study, the AOM/DSS-induced mouse model of colorectal cancer was successfully established and a significant increase in PLAC8 expression during tumor formation, suggesting that PLAC8 may play an important role in the inflammation-cancer transition in CRC. We attempted to generate PLAC8 knockout mice using conventional gene targeting methods, but the resulting mice were non-viable. Future studies will employ conditional knockout strategies to circumvent this embryonic and validate metastasis-microenvironment links.

Numerous studies have identified PLAC8 as an oncogene associated with poor prognosis in various types of cancers. As a centrosomal oncoprotein, PLAC8 plays a pivotal role in driving colorectal cancer progression by promoting tumor growth and metastasis (21). A total of 78 CRC cases were included in this study. PLAC8 protein expression in tumor tissues and adjacent normal tissues was analyzed, and its association with clinicopathological characteristics and patient prognosis was evaluated. Results demonstrated that PLAC8 was significantly overexpressed in CRC tissues and negatively correlated with patient prognosis. Univariate Cox regression analysis confirmed that high PLAC8 expression, advanced T, N, and M stages, poor tumor differentiation, and elevated CA199 levels were significantly associated with advanced disease stage and poor prognosis. Moreover, multivariate Cox regression analysis identified high PLAC8 expression, higher TNM stage, and poor tumor differentiation as independent prognostic risk factors for CRC. In colorectal cancer, this study further confirms the oncogenic role of PLAC8 and demonstrated its association with tumor TNM stage, suggesting that PLAC8 may play an important role in tumor invasion and metastasis.

PLAC8 has been identified as a key regulator in the progression of various cancers by inducing tumorigenesis, modulating immune responses, promoting chemoresistance, and facilitating metastasis (22). It has been reported to activate the AKT signaling pathway, thereby enhancing sorafenib resistance in hepatocellular carcinoma (HCC) cells (23). PLAC8 promotes epithelial-mesenchymal transition (EMT) and cervical cancer progression (24), drives lung cancer cell proliferation via the Wnt/β-catenin signaling pathway (25), and modulates tamoxifen sensitivity through the MAPK/ERK signaling pathway (26). Furthermore, PLAC8 inhibits apoptosis, leading to radiotherapy resistance in nasopharyngeal carcinoma (NPC) cells (27), and suppresses autophagy, contributing to doxorubicin resistance in breast cancer and enhancing proliferation and EMT in NPC cells (28, 29). Additionally, PLAC8 has been identified as a key molecule in reshaping the tumor microenvironment of clear cell renal cell carcinoma (ccRCC), negatively impacting proliferation, invasion, migration, and immunotherapy efficacy (30). Although many studies suggest that PLAC8 functions as an oncogene, others report a tumor-suppressive role. For example, PLAC8 inhibits oral squamous cell carcinogenesis and EMT via the Wnt/β-catenin and PI3K/Akt/GSK3β signaling pathways (31). In this study, bioinformatics analysis revealed that PLAC8 might be involved in cytokine-related pathways and platinum-based drug resistance. However, subsequent biological functional experiments showed that PLAC8 expression had no significant impact on proliferation, colony formation, or oxaliplatin resistance in colorectal cancer cell lines. Notably, PLAC8 overexpression markedly enhanced cell migration. Further analysis of cytokine-related pathways demonstrated that PLAC8 robustly activated the AKT signaling pathway, while having minimal effects on NF-κB, ERK, and STAT3 pathways. EMT-related markers, including N-cadherin and Snail1, were significantly upregulated, while E-cadherin expression remained unchanged, suggesting that PLAC8 might promote colorectal cancer cell migration by activating the AKT signaling pathway and inducing EMT. Rescue experiments using the AKT pathway activator SC79 and inhibitor Capivasertib further confirmed that PLAC8 enhances EMT, migration, and invasion through the AKT signaling pathway. This result was also confirmed in another study (32).

PLAC8 has been shown in numerous studies to promote tumorigenic processes, including proliferation, metastasis, chemoresistance, and radiotherapy resistance, via classic signaling pathways such as AKT, ERK, and Wnt. However, relatively few studies have focused on the regulation of PLAC8 expression itself. Limited evidence suggests that miR-1228-3p, miR-664b-3p, and UFM1-mediated ubiquitination may play a role in regulating PLAC8 expression (23, 33, 34). PLAC8 also functions as a core downstream effector of the Id1-c-Myc axis, sustaining colorectal cancer stemness, promoting self-renewal, and conferring chemoresistance by activating Wnt/β-catenin and Shh signaling pathways (35). In this study, bioinformatics analysis identified the five genes most closely associated with PLAC8 expression as CCL28, CXCL1, ITK, JAK2, and STAT3. Correlation analyses derived from databases are inherently limited and lack direct biological significance; they primarily offer guidance for research direction selection but must be experimentally validated. Subsequent literature review and experimental validation revealed that STAT3, a transcription factor in the JAK-STAT3 signaling pathway, can directly bind to the PLAC8 promoter region, enhancing its activation and expression. CCL28, a mucosal-associated epithelial chemokine, is known to recruit various immune cells, modulate immune cell activation and chemotaxis in the tumor microenvironment, and exhibit tumor-suppressive functions (36). However, our study found that CCL28 acts as an upstream factor in activating the STAT3 signaling pathway and mediating PLAC8 expression, thereby promoting colorectal cancer cell migration. CUT&Tag assay confirmed that STAT3 directly bounding of STAT3 to the PLAC8 gene promoter region and indicating transcriptional regulation (**Figure 4F**). The STAT3 pathway inhibitor Ruxolitinib was shown to suppress PLAC8 expression by inhibiting STAT3 signaling, leading to a significant reduction in colorectal cancer cell migration. Rescue experiments demonstrated that this suppression could be reversed by CCL28, suggesting a critical role for CCL28 in this regulatory axis. These findings suggest that, in this context, CCL28 may exhibit oncogenic properties, which contrasts with other studies suggesting a tumor-suppressive role. This disparity highlights the complexity of CCL28’s function, likely influenced by multiple factors in the tumor microenvironment.

PLAC8 and CCL28 have emerged as key regulators in the inflammation-to-cancer transition of CRC, particularly in shaping the tumor microenvironment (TME) and immune modulation. Recent studies have shown that both are involved in tumor immune evasion, immune cell infiltration, and immunotherapy response. PLAC8 influences CRC cell proliferation, migration, and invasion by modulating the TME. In clear cell renal cell carcinoma (ccRCC), its high expression correlates with poor prognosis and affects the cell cycle, ROS pathways, and immune infiltration, thereby impacting immunotherapy outcomes (30). In triple-negative breast cancer, PLAC8 regulates PD-L1 expression, suggesting a potential immunotherapy target (34). CCL28, similarly, has been shown to promote immune evasion. In CRC, SPDEF-induced CCL28 expression inhibits M2 macrophage polarization, limiting immune suppression (37). In lung cancer, CCL28 recruits regulatory T cells (Tregs), which may reduce immunotherapy efficacy (38). Preliminary data from our laboratory indicate a positive correlation between PLAC8 expression and T cell-mediated cytotoxicity. Together, these findings highlight PLAC8 and CCL28 as critical modulators of the TME and potential targets for cancer immunotherapy. Further studies on their mechanisms and immune interactions may help optimize therapeutic strategies across tumor types.

**Figure 5:**
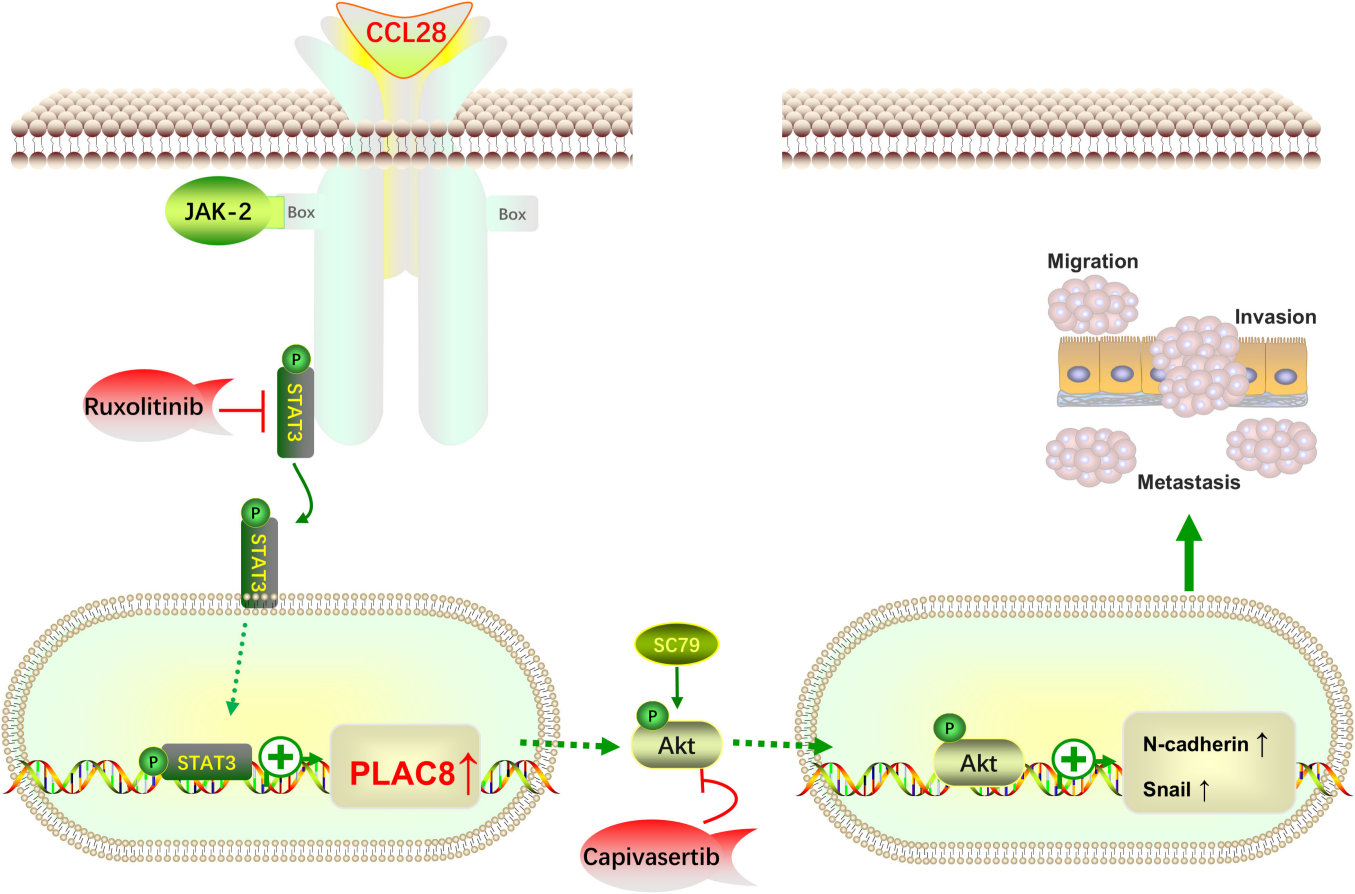
Schematic illustrating how the CCL28-STAT3-PLAC8 axis promotes EMT and enhances CRC invasion and metastasis via AKT signaling activation.

In conclusion, this study integrates clinical cases, animal models, cell-based experiments, and bioinformatics analyses to, for the first time, propose that CCL28 promotes PLAC8 expression through activation of the STAT3 signaling pathway. PLAC8 subsequently activates the AKT signaling pathway, driving EMT and facilitating colorectal cancer progression (**Figure 5**). PLAC8 is identified as a key factor in this process, playing a critical role in colorectal cancer cell migration and invasion. While the findings are primarily based on cellular experiments, further validation using larger cohorts of clinical samples is necessary to strengthen the evidence. Transient transfection sufficiently established acute signaling/phenotypic causality. Future *in vivo* and chronic interaction studies will employ lentiviral, shRNA, or CRISPR systems. Additionally, as a part of the inflammation-cancer transition, the role of immune cells and other components of the tumor microenvironment warrants further investigation. Our study identifies PLAC8 as a promising target for therapeutic intervention of colorectal cancer, offering new insights into its role in tumor progression and the broader context of the inflammation-cancer axis.

## Data Availability

The datasets presented in this study can be found in online repositories. The names of the repository/repositories and accession number(s) can be found in the article/**Supplementary Material**.
