# Supplementary material for: Targeting the CCL28-STAT3-PLAC8 axis to suppress metastasis and remodel tumor microenvironment in colorectal cancer
Source: Front Immunol. 2025 Oct 1;16:1610540. doi: 10.3389/fimmu.2025.1610540 (PMC12521221; doi:10.3389/fimmu.2025.1610540)
Supplement: Supplementary file 1 [file DataSheet1.docx]

**Supplementary Table 1 The primer sequences used in this study are as follows (5′→3′ orientation)**

| **Application** | **sequences** | **sequences** |
| --- | --- | --- |
| **Q-PCR** |  |  |
| PLAC8 | S: AACAAGCGTCGCAATGAGGA | A: AGTACGCATGGCTCTCCTTCTGT |
| GAPDH | S: GGAAGCTTGTCATCAATGGAAATC | A: TGATGACCCTTTTGGCTCCC |
| **CUT-Tag** |  |  |
| PLAC8-1 | F: ACCAAACCAGAATGAAG | R: TTGACTGAAATCTCCTGT |
| PLAC8-2 | F: CTTGTGCTAAGTGACATAT | R: CTGAGGTTGACTGAAATC |
| **PLAC8-siRNA** |  |  |
| siRNA1 | Sense: GGUCCCGGUCCGGCCCCCCTT | Antisense: GGGGGGCCGGACCGGGACCTT |
| siRNA2 | Sense: UCUUUGCUGUCCUCAUUGUTT | Antisense: ACAAUGAGGACAGCAAAGATT |
| siRNA3 | Sense: AGUCUGUCUCUGUGGCACATT | Antisense: UGUGCCACAGAGACAGACUTT |

| Characteristics | Count (n) | Characteristics | Count (n) |
| --- | --- | --- | --- |
| Age (year) |  | T staging |  |
| <60 | 34 | T1 | 5 |
| ≥60 | 44 | T2 | 9 |
| Gender |  | T3 | 17 |
| Female | 30 | T4 | 47 |
| Male | 48 | N staging |  |
| TNM staging |  | N0 | 31 |
| I | 11 | N1 | 31 |
| II | 18 | N2 | 16 |
| III | 33 | M staging |  |
| IV | 16 | M0 | 66 |
| Differentiation |  | M1 | 12 |
| High | 3 | Location |  |
| Moderate | 51 | Colon | 13 |
| Low | 24 | Rectum | 65 |
| CA125 |  | CEA |  |
| ≥35U/mL | 7 | ≥5ng/mL | 25 |
| <35U/mL | 71 | <5ng/mL | 50 |
| CA199 |  |  |  |
| ≥37U/mL | 23 |  |  |
| <37U/mL | 52 |  |  |

**Supplementary Table 2 Clinicopathological characteristics of 78 Colorectal adenocarcinoma patients in this study**

**Supplementary Table 3 Comparison of clinicopathological characteristics in high and low PLAC8 groups**

| Characteristics | High (n) | Low (n) | *P* value |
| --- | --- | --- | --- |
| Age (year) |  |  | 0.512 |
| <60 | 5 | 33 |  |
| ≥60 | 9 | 44 |  |
| Gender |  |  | 0.512 |
| Female | 6 | 20 |  |
| Male | 8 | 40 |  |
| TNM staging |  |  | 0.428 |
| I | 2 | 9 |  |
| II | 1 | 17 |  |
| III | 8 | 25 |  |
| IV | 3 | 13 |  |
| Differentiation |  |  | 0.512 |
| High | 0 | 2 |  |
| Moderate | 10 | 42 |  |
| Low | 4 | 20 |  |
| T staging |  |  | 0.572 |
| T1 | 0 | 5 |  |
| T2 | 2 | 7 |  |
| T3 | 2 | 15 |  |
| T4 | 10 | 37 |  |
| N staging |  |  | 0.182 |
| N0 | 3 | 28 |  |
| N1 | 6 | 25 |  |
| N2 | 5 | 11 |  |
| M staging |  |  | 0.489 |
| M0 | 11 | 55 |  |
| M1 | 3 | 9 |  |
| Location |  |  | 0.187 |
| Colon | 10 | 55 |  |
| Rectum | 4 | 9 |  |
| CA125 |  |  | 0.443 |
| ≥35U/mL | 2 | 5 |  |
| <35U/mL | 12 | 59 |  |
| CA199 |  |  | 0.008 |
| ≥37U/mL | 8 | 15 |  |
| <37U/mL | 5 | 47 |  |
| CEA |  |  | 0.666 |
| ≥5ng/mL | 5 | 20 |  |
| <5ng/mL | 8 | 42 |  |

**Supplementary Table 4 Cox univariate analysis of clinicopathological characteristics and PLAC8 expression**

| Characteristics | Count (n) | HR | *P* value |
| --- | --- | --- | --- |
| Age (year) |  | 1.25 | 0.504 |
| <60 | 34 |  |  |
| ≥60 | 44 |  |  |
| Gender |  | 1.61 | 0.144 |
| Female | 30 |  |  |
| Male | 48 |  |  |
| TNM staging |  | 3.33 | <0.001 |
| I | 11 |  |  |
| II | 18 |  |  |
| III | 33 |  |  |
| IV | 16 |  |  |
| Differentiation |  | 2.36 | 0.006 |
| High | 2 |  |  |
| Moderate | 52 |  |  |
| Low | 24 |  |  |
| PLAC8 expression |  | 2.36 | 0.021 |
| High | 14 |  |  |
| Low | 64 |  |  |
| CA125 |  | 2.23 | 0.10 |
| ≥35U/mL | 7 |  |  |
| <35U/mL | 71 |  |  |
| CA199 |  | 2.15 | 0.021 |
| ≥37U/mL | 23 |  |  |
| <37U/mL | 52 |  |  |
| T staging |  | 3.04 | 0.001 |
| T1 | 5 |  |  |
| T2 | 9 |  |  |
| T3 | 17 |  |  |
| T4 | 47 |  |  |
| N staging |  | 4.44 | <0.001 |
| N0 | 31 |  |  |
| N1 | 31 |  |  |
| N2 | 16 |  |  |
| M staging |  | 6.13 | <0.001 |
| M0 | 66 |  |  |
| M1 | 12 |  |  |
| Location |  | 1.63 | 0.311 |
| Colon | 13 |  |  |
| Rectum | 65 |  |  |
| CEA |  | 0.93 | 0.83 |
| ≥5ng/mL | 25 |  |  |
| <5ng/mL | 50 |  |  |
