# Supplementary material for: Targeting the CCL28-STAT3-PLAC8 axis to suppress metastasis and remodel tumor microenvironment in colorectal cancer
Source: Front Immunol. 2025 Oct 1;16:1610540. doi: 10.3389/fimmu.2025.1610540 (PMC12521221; doi:10.3389/fimmu.2025.1610540)
Supplement: Supplementary file 2 [file Presentation1.pptx]

## Slide 1
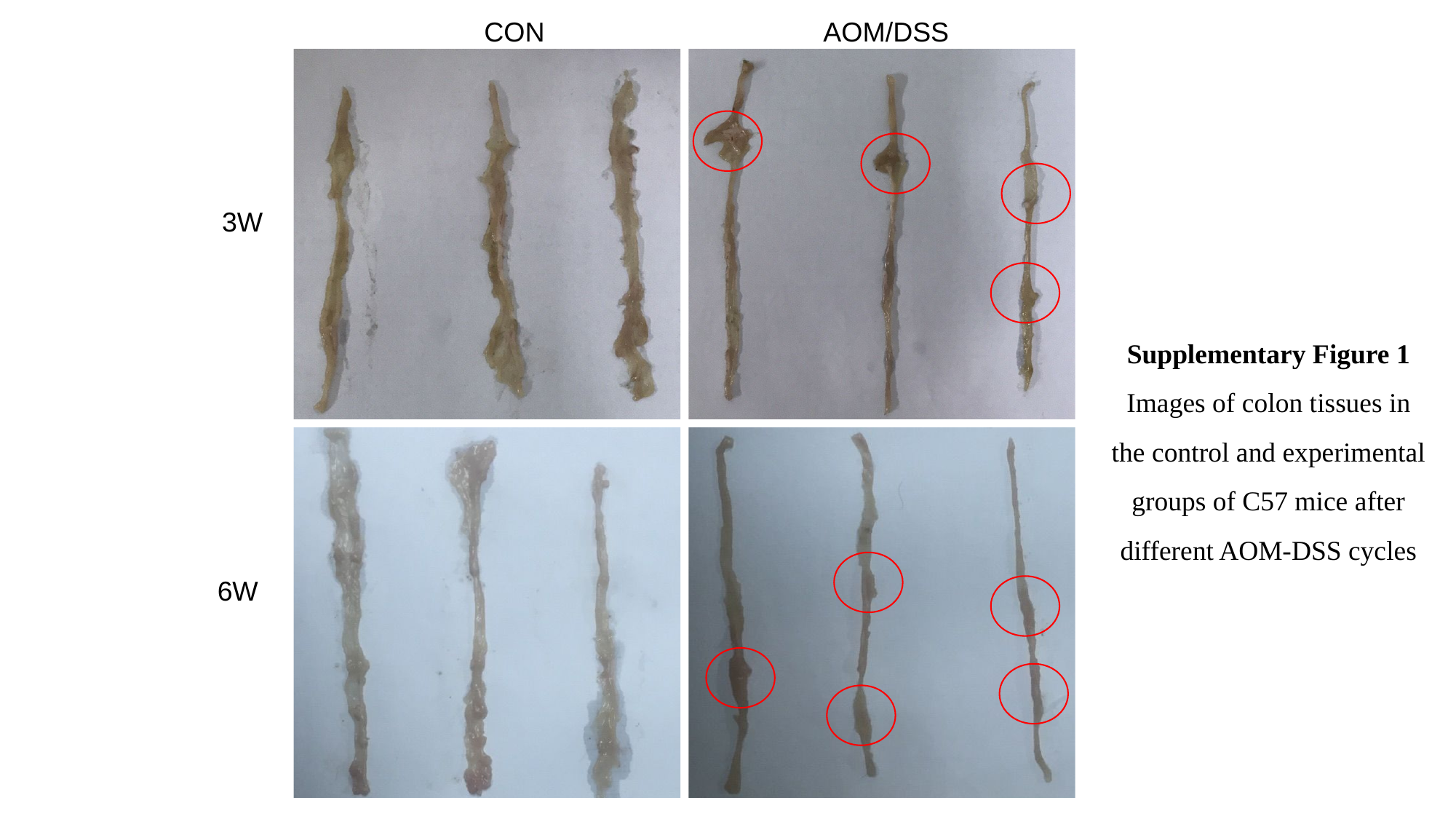

CON
AOM/DSS
3W
6W
Supplementary Figure 1 Images of colon tissues in the control and experimental groups of C57 mice after different AOM-DSS cycles
